# Unmasking Tuberculosis Pericarditis: The Role of Xpert MTB/RIF in Early Diagnosis and Treatment—A Case Report

**DOI:** 10.1155/crdi/2518518

**Published:** 2026-07-20

**Authors:** Saumya Nanda, Tharun Shyam, Prabal K. C., Oluchi Ndulue, Daniel Jacobs, Armando Seitllari, Shaurya Sharma, Yu Shia Lin, Vijay Shetty

**Affiliations:** ^1^ Internal Medicine, Maimonides Medical Center, Brooklyn, New York, USA, maimonidesmed.org

**Keywords:** cardiac tamponade, case report, tuberculous pericarditis, Xpert MTB/RIF

## Abstract

Tuberculous pericarditis (TBP) is an uncommon but serious form of extrapulmonary tuberculosis that remains diagnostically challenging due to its nonspecific clinical features and the limited sensitivity of conventional methods such as smear microscopy, culture, and histopathology. We describe an 80‐year‐old man presenting with recurrent fever and weakness, found to have a large pericardial effusion with tamponade physiology requiring emergent pericardial window drainage. Pericardial fluid analysis demonstrated an exudative, lymphocyte‐predominant effusion with elevated lactate dehydrogenase and low glucose levels, but initial acid‐fast bacillus smear, histopathology, and sputum studies were negative. Xpert MTB/RIF testing performed on pericardial fluid detected *Mycobacterium tuberculosis*, enabling early initiation of antitubercular therapy prior to culture confirmation. This case underscores the importance of considering TBP in patients with unexplained pericardial effusion, particularly in those with epidemiologic risk factors, and highlights the diagnostic value of Xpert MTB/RIF as a rapid and highly specific molecular assay that can expedite diagnosis and improve clinical outcomes.

## 1. Background

Tuberculosis pericarditis (TBP) is a form of extrapulmonary tuberculosis that presents with significant diagnostic challenges. Delayed diagnosis can lead to severe complications and is associated with high morbidity and mortality. Conventional diagnostic techniques, such as mycobacterial culture and histopathology, often take weeks to yield conclusive results, delaying the initiation of appropriate treatment. Xpert MTB/RIF, a rapid molecular test, offers a faster and more reliable alternative, enabling the early detection of *Mycobacterium tuberculosis* in pericardial fluid while simultaneously identifying rifampicin resistance. This case report was prepared in accordance with the CAse REport (CARE) guidelines (Supporting Materials).

## 2. Case Presentation

An 80‐year‐old male with a medical history of hypothyroidism and benign prostatic hyperplasia presented to the emergency department with an eight‐day history of intermittent fever and progressive weakness. He denied weight loss, night sweats, or a prior history of tuberculosis. The patient had emigrated from Pakistan in the 1980 s, worked in construction, and owned a pet parrot. He had no known medication or food allergies.

In the week preceding this presentation, the patient visited the emergency department twice for similar complaints and underwent comprehensive infectious workups, which were unremarkable. On his most recent visit, he was prescribed cefdinir for a presumptive urinary tract infection, which he had taken for 4 days prior to his current presentation. His regular medications included dutasteride, levothyroxine, omega‐3 fatty acids, and tamsulosin.

On arrival, the patient was febrile with a temperature of 103.1 °F, a heart rate of 117 beats per minute, a blood pressure of 141/85 mmHg, a respiratory rate of 18 breaths per minute, and an oxygen saturation of 93% on ambient air. His body mass index (BMI) was 26. Physical examination revealed distant heart sounds without any other significant findings.

Laboratory investigations revealed a leukocyte count of 10.6 K/μL, hemoglobin of 13.3 g/dL, LDH of 198 IU/L, C‐reactive protein of 37.9 mg/dL, erythrocyte sedimentation rate of 93 mm/hr, and thyroid‐stimulating hormone of 6.91 µIU/mL. A 4th‐generation HIV test was nonreactive (Table 1).

**TABLE 1:** Serum investigations.

Test	Result
Leukocyte count	10.6 K/uL
Hemoglobin	13.3 gm/dL
LDH	198 IU/L
C‐reactive protein	37.9 mg/dL
Erythrocyte sedimentation rate	93 mm/hr
TSH	6.91 ulU/mL
HIV (4th generation)	Nonreactive

**FIGURE 1:**
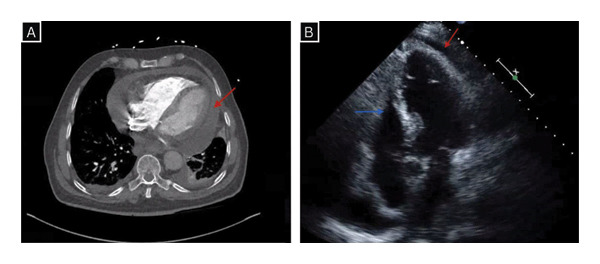
(A) Chest CT showing pericardial effusion (red arrow) with pericarditis and mediastinal lymphadenopathy. (B) Transthoracic echocardiogram (TTE) illustrating circumferential pericardial effusion (red arrow) with signs of right ventricular (RV) (blue arrow) and right atrial (RA) collapse.

A chest X‐ray showed no abnormalities. CT imaging revealed a large, circumferential pericardial effusion with pericardial enhancement and mediastinal lymphadenopathy (Figure 1A). Transthoracic echocardiography (TTE) demonstrated a large pericardial effusion with right atrial and right ventricular collapse, consistent with cardiac tamponade (Figure 1B). This necessitated an urgent pericardial window procedure with drainage of the effusion. Following the procedure, approximately 800 mL of serosanguineous pericardial fluid was drained, and the patient’s fever subsided.

The QuantiFERON‐TB Gold assay was positive. Sputum AFB smears and cultures and sputum Xpert MTB/RIF assays were all negative. Additional laboratory testing was negative for other respiratory viruses, Cytomegalovirus, Coxsackie B virus, Bartonella species, Mycoplasma, and Chlamydia, and the vasculitis workup was also unremarkable.

Pericardial fluid analysis revealed an exudative fluid with elevated LDH, low glucose, and lymphocytic predominance (Table 2). Histopathology of the pericardium demonstrated chronic inflammation and granulation tissue without granulomas. AFB smear was negative. A 5‐mL sample of pericardial fluid was submitted for Xpert MTB/RIF testing and returned positive for *M. tuberculosis*, with no evidence of rifampin resistance on *rpoB* gene analysis. The specimen was subsequently cultured in both liquid broth and solid media for isolation and identification of *M. tuberculosis* complex.

**TABLE 2:** Pericardial fluid investigations.

Pericardial fluid (PF) test	Result
Color/appearance	Red/cloudy
WBC	378 cells/uL
RBC	24,000/uL
Lymphocytes	97%
Monocytes	3%
LDH	1,543 IU/L
Glucose	20 mg/dL
Total protein	5.3 gm/dL
Acid‐fast bacilli (AFB) smear	Negative
Malignant cells	Negative

The patient was promptly initiated on antitubercular therapy with isoniazid, rifampin, pyrazinamide, and ethambutol (RIPE). Growth of acid‐fast bacilli (AFB) was detected in the broth after 15 days. At 3 weeks, the pericardial fluid AFB culture confirmed the growth of the *M. tuberculosis* complex. Final species identification was confirmed by matrix‐assisted laser desorption ionization–time of flight (MALDI‐TOF) mass spectrometry. The patient tolerated the therapy well, and a follow‐up echocardiogram 3 months later showed complete resolution of the pericardial effusion.

## 3. Discussion

TBP accounts for less than 5% of all pericarditis cases in North America and Europe, where 80%–90% of the cases are classified as idiopathic or presumed viral [1, 2]. In contrast, in tuberculosis‐endemic regions such as sub‐Saharan Africa, tuberculosis is the leading cause of pericarditis, responsible for approximately 70% of pericardial effusions, and is the most common cause of constrictive pericarditis [1, 2]. Constrictive pericarditis is among the most severe complications of TBP, developing in approximately 20%–30% of the affected individuals even with optimal antitubercular therapy [3, 4]. Given the potential for such serious sequelae, early and accurate diagnosis of TBP is essential to initiate appropriate treatment and reduce the risk of progression to constriction. However, early diagnosis remains challenging, as TBP often mimics other pericardial diseases, including viral, autoimmune, and neoplastic conditions.

In the present case, the initial differential diagnosis was broad and included malignancy, viral infections, autoimmune disorders, fungal etiologies, and tuberculosis. Diagnostic uncertainty was compounded by the paucibacillary nature of extrapulmonary tuberculosis, which limits the sensitivity of conventional tests such as AFB smear microscopy [5]. Sputum cultures for Mycobacterium tuberculosis are positive in only 10%–55% of cases [6]. In our patient, several features, including the absence of constitutional symptoms (e.g., weight loss and night sweats), a normal chest radiograph, negative pericardial fluid AFB smears, and a lack of granulomatous inflammation on histopathology, argued against TBP. Moreover, the resolution of fever following pericardial window drainage further lowered clinical suspicion, delaying diagnosis.

Despite these findings, the biochemical profile of the pericardial fluid was suggestive of a nonviral inflammatory process. The effusion was exudative, with elevated protein levels, lymphocytic predominance, elevated LDH, and low glucose—features classically associated with TBP. In light of these findings, Xpert MTB/RIF testing was performed on the pericardial fluid. This rapid, PCR‐based assay detects *M. tuberculosis* DNA and rifampicin resistance within 2 hours [7]. Current clinical guidelines from the World Health Organization (WHO) and the American Thoracic Society (ATS) support the conditional use of Xpert MTB/RIF for extrapulmonary tuberculosis, including TBP, to facilitate timely diagnosis and early initiation of treatment [8, 9].

Xpert MTB/RIF, though not FDA‐approved specifically for TBP, remains instrumental due to its speed and minimal technical requirements. It reduces the diagnostic delay inherent in mycobacterial cultures, which often take weeks, and provides early insight into drug resistance patterns, allowing for timely and tailored therapeutic approaches. A recent systematic review and meta‐analysis by Andrianto A et al. assessed the diagnostic accuracy of Xpert MTB/RIF for TBP using data from nine studies encompassing 581 patients. The assay demonstrated high specificity (99.4%) but only moderate sensitivity (67.6%) for detecting *M. tuberculosis* in pericardial fluid. Though its sensitivity for pericardial fluid TB detection ranges from 30% to 70%, it remains a critical highlight of the continued need for more sensitive diagnostic approaches, especially in smear‐negative or paucibacillary cases [10]. Another recent meta‐analysis by Yu G et al. evaluated the diagnostic performance of Xpert MTB/RIF for TBP and reported a pooled sensitivity and specificity of 65% and 99%, respectively, when compared to a composite reference standard (CRS), with an AUC of 0.99 [11]. Consequently, a positive result serves as a highly reliable rule‐in test, whereas a negative result cannot definitively exclude disease.

## 4. Conclusion

This case highlights the importance of maintaining a high index of suspicion for tuberculosis in patients with pericarditis, particularly those with relevant epidemiological exposures, even when initial diagnostic studies are inconclusive. The use of Xpert MTB/RIF enables faster diagnosis and treatment, which is pivotal in preventing TBP complications and improving outcomes.

## Funding

The authors did not receive any funding from any organization for the submitted work.

## Disclosure

The abstract of the manuscript was presented at New York Chapter of the American College of Physicians Annual Scientific Meeting in October 2024. Reference: Final Poster Book 2024:85. https://www.nyacp.org/files/Final_Poster_Book_2024(1).pdf.

## Consent

Informed consent for publication of this case report was obtained.

## Conflicts of Interest

The authors declare no conflicts of interest.

## Supporting Information

Additional supporting information can be found online in the Supporting Information section.

## Supporting information


**Supporting Information** The CARE checklist (CARE‐checklist‐English‐2013_2.pdf) is available as supporting material.

## Data Availability

Data sharing is not applicable to this article as no datasets were generated or analyzed during the current study.
